# Quantitative Properties of the Macro Supply and Demand Structure for Care Facilities for Elderly in Japan

**DOI:** 10.3390/ijerph14121489

**Published:** 2017-12-01

**Authors:** Tatsuya Nishino

**Affiliations:** Faculty of Environmental Design, College of Science and Engineering, Kanazawa University, Kanazawa 920-1192, Japan; tan378@se.kanazawa-u.ac.jp

**Keywords:** the elderly, quantitative index, group living facilities, community-based multi-care facility, the Long-Term Care Insurance Act

## Abstract

As the Asian country with the most aged population, Japan, has been modifying its social welfare system. In 2000, the Japanese social care vision turned towards meeting the elderly’s care needs in their own homes with proper formal care services. This study aims to understand the quantitative properties of the macro supply and demand structure for facilities for the elderly who require support or long-term care throughout Japan and present them as index values. Additionally, this study compares the targets for establishing long-term care facilities set by Japan’s Ministry of Health, Labor and Welfare for 2025. In 2014, approximately 90% of all the people who were certified as requiring support and long-term care and those receiving preventive long-term care or long-term care services, were 75 years or older. The target increases in the number of established facilities by 2025 (for the 75-years-or-older population) were calculated to be 3.3% for nursing homes; 2.71% for long-term-care health facilities; 1.7% for group living facilities; and, 1.84% for community-based multi-care facilities. It was revealed that the establishment targets for 2025 also increase over current projections with the expected increase of the absolute number of users of group living facilities and community-based multi-care facilities. On the other hand, the establishment target for nursing homes remains almost the same as the current projection, whereas that for long-term-care health facilities decreases. These changes of facility ratios reveal that the Japanese social care system is shifting to realize ‘Ageing in Place’. When considering households’ tendencies, the target ratios for established facilities are expected to be applied to the other countries in Asia.

## 1. Introduction

This study aims to understand the quantitative properties of the macro supply and demand structure for facilities for elderly persons requiring support and long-term care throughout Japan, and present them as index values.

One of the great human success stories in the last few decades has been the marked increase in longevity in many societies across the globe [1]. Population ageing transcends the divide between developed and developing countries, because every country hopes to foster independent living and quality of life, regardless of life expectancy [2]. In addition, an increasingly ageing population provides a challenge to ageing countries in terms of meeting the needs of an ageing population and creating a policy environment [3].

One of the main issues in an ageing society has been the provision of housing for the elderly [4,5]. For example, in Denmark, which is known for its highly developed social care system for the aged, formal care was introduced as an alternative to informal care by families [6]. They have developed new attitudes towards old age and new ways of housing and providing services to the elderly since 1980. Moreover, their policy has encouraged the elderly to stay in their own homes for as long as possible [7]. Alternatively, in the Netherlands, a de-linking system of housing and care has been developed, in order to make elderly people more autonomous and to force care providers to be more customer-oriented [8].

In Asia, most countries will be ‘aged societies’ by 2050, a phenomenon known as ‘Ageing Asia’ [9]. While currently in Asian countries the elderly has been, and remain, primarily cared for by their own families [10,11], previous studies have pointed out the need to change from traditional family support to public care systems [12,13,14]. In accordance with this process of change, Hiroi showed three phases of social security system development using the notion of health transition [15]. In the first phase of health transition, the main problem is infectious diseases. Therefore, the key issue is public health policy, and programs should be financed by public taxation. In the second phase, disease structure changes from the group level to the personal level, such as chronic diseases. Therefore, the insurance system is mainly selected to finance social security in this phase. Medical services and hospitalizations are mainly used. Japan went through this phase in 1960–1970. In the third phase, the need for elderly care becomes high. The elderly need not only medical services, but also care services. Therefore, there are two shifts: one from medical services to social welfare services, and the other from institutional service to in-home service. Integration of these shifts becomes the main topic of system design.

Ohizumi applied Hiroi’s development process model to Asian countries and categorized them on the basis of their social security systems and challenges, economic development, and social change [16]. The countries in the first group (Japan, South Korea, Taiwan, Hong Kong, and Singapore) have highly developed economies, low birth rates, and aging populations. Their national social security systems cover all of their citizens, but the main challenge is to keep the systems sustainable as the populations age. The countries in the second group (Malaysia, Thailand, Philippines, Indonesia, and China) have been undergoing industrialization. Their social security systems cover only the employed. Their challenge is to establish national medical insurance systems and pension systems that cover farmers and the self-employed. The countries in the third group (Vietnam, India, Laos, Cambodia, and Myanmar) have started to industrialize relatively recently. Their systems cover only public employees and military personnel, and systems that are covering private sector employees are under construction.

As the Asian country with the most aged population, Japan has been modifying its social welfare system since 1989 [17]. In 2000, the Japanese social care vision turned towards keeping the elderly with care-needs in their own homes with proper formal care services, by introducing ‘Kaigo Hoken’ (The Long-Term Care Insurance Act). This vision seems to be following the ageing policies of advanced countries, such as Denmark. These polices are based on the notion of ‘Aging in Place’. Elderly people normally prefer to live in familiar residences, particularly their homes, where they have memories and that have special meanings attached [18].

Although Japan follows the “ageing advanced” countries in its care vision, the Japanese elderly care system can become an example of good practice in an ageing society for the following reasons. First, on the one hand, the characteristic of Japanese care service centers is that their use is restricted to only those elderly people who need care services [19]. The elderly who do not need care services go to community centers [20]. On the other hand, in Aarhus in Denmark, for example, there are senior centers where every elderly person can go and receive care services if he/she needs them [21]. Secondly, Hiroi says Japan’s experience can offer a useful and common model from the viewpoint of social security system design for countries that have started industrializing and rapidly aging [15].

In Japan, the notion of ‘the Community-based Integrated Care System’ was introduced into public law as the Long-Term Care Insurance Act, in 2013 [22]. It is a system that provides housing; medical care; long-term care; prevention services; and, livelihood support in an integrated manner in communities (which assume the approximate range of a junior high school district) (Table 1, [23]). Table 2 also shows a detailed menu of long-term care and preventative long-term care services and their expense percentages [24]. This system aims to enable people to continue to live in their home towns to the end of their lives with a sense of security once they are in serious need of long-term care.

According to the medium-range projections of the National Institute of Population and Social Security Research [25], Japan will continue to see a net increase in its elderly population, that is, persons 65 years or older, until the year 2042. For this reason, plans for increases in elderly care facilities and services will be likely to continue in the future. However, the elderly care plans that each insurer (a municipality or organizations affiliated with it) makes only cover a short-term period of three years. Care facilities, once they are built, have a life of about forty years. So it is important that we consider the peaks in the absolute numbers of the elderly population and establish long-term elderly care services and facilities plans on this basis. 

In such long-term plans, when the future needs for the various services and facilities for taking care of the elderly are calculated, one basis for that calculation will be an estimate of the total number of the elderly population. It would be useful if a projection of the amount of care services and the number of facilities needed was based on an estimate of the size of the elderly population. However, this is not easily done. For example, there are no reference values that are available for the rate of facility development for the population of elderly people. In Japan as a whole, there are numerical objectives for the establishment to be set up for various facility services in the year 2025, which is the year when the last of the baby boomers will join the latter stage of the elderly population. However, these numbers are not expressed in terms of an elderly care establishment rate that each insurer can apply [26]. In addition, there are regulations on the total number of care facilities, such as nursing homes, for the aged. It has also been pointed out that there are cases in which, depending on the municipality, resistance to increases in the burden of public assistance and in the amount of compensation for elderly care services has occurred. In such cases, reactions to the approval of the establishment of elderly care facilities have been negative [27]. As a result, depending on the location (the insurer), disparities have arisen in the rate of establishment of an establishment of services and facilities for elderly care.

Given the above objectives, the purpose of this research has been to index and quantify the number of users of each type of elderly care facility for each insurer nationwide in 2010. The process that is used to do this is as follows:

(a) First, the number nation-wide of elderly who require support or care was determined.
(b) Next, the quantitative objectives for facility establishment given in the national reform model for 2025 were indexed. These objectives were also compared with the indices that were related to the number of facility users.
(c) Finally, after quantifying the data above on the number of users in regard to a given insurer, the study focused on the possibility of attaining the facility establishment rate objectives for the year 2025.

Regarding previous research, Osakaya et al. (1996) have predicted the likely demand for facilities for the elderly in three cities in Hokkaido (Muroran, Noribetsu, and Date) [28]. However, since then, new types of facilities have been introduced, due to the introduction of the Long-Term Care Insurance system, and the facilities system has changed. In addition, Sonoda et al. (1993) studied an urban development area (the Suwa District in Tama New Town) [29,30]. Based on estimates of the demand for facilities for the elderly, they have been studying a plan for the re-allocation of such facilities. However, during the past fifteen years or so, there has been almost no quantitative research on the demand for services for the elderly in the field of facility planning. For this reason, previous research has not included any nationwide comparisons regarding the rate of establishment facilities for the elderly, and no reference values have been specified. By displaying these values from estimates of the elderly population, rough numbers of the facilities for the elderly that will be needed in the future can be easily calculated. Hence, the significance of this research. In addition, depending on the approach of each insurer, the disparities that are described above will appear. Without a nationwide index, it is impossible to make comparisons. As a result of this research, it is possible to grasp the relative position of the level of facility establishment for each insurer. 

## 2. Materials and Methods

**Figure 1:**
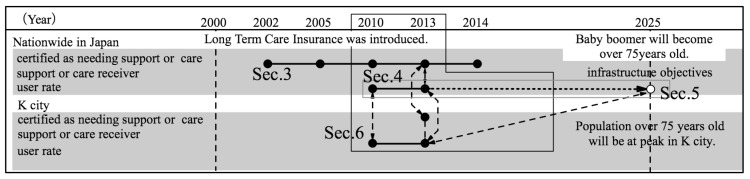
Composition of this paper.

This research is composed of four parts (Figure 1) as follows.

Determination of the number of elderly nationwide who require support or care (Section 3).

Indexing of the number of users of elderly care insured facilities, for each nationwide insurer in 2010 (Section 4).

Comparison of the above indices and the facility establishment rate objectives in the reform model for the year 2025 (Section 5).

Determination of the above quantitative situation for a given insurer and study of the possibility of applying to the insurer the facility establishment rate objectives in the reform model for the year 2025 (Section 6).

### 2.1. Points of Analysis and Methods of Conducting the Nationwide Survey

This research uses administrative data because they were the only data that could explain the nationwide situation, the changes in the elderly population, and the number of persons needing and certified for elderly support and care. The annual data are comparable because statistical information formats have not changed since 2002, and they are publicly available and free to access via websites. They provide almost completely reliable numbers for persons needing and certified for elderly support and care and persons receiving preventive care services or nursing care services, because they are based on service fees. However, we have to be aware that the total number of users is different from the number of actual users annually. For example, a user of a nursing home may be hospitalized and then enter the nursing home again after getting out of the hospital. In such a case, two persons will be counted as being in the nursing home. Including this type of example, the data that can be gathered about users are the only data currently available. Thus, the number of users calculated will be slightly more than the actual number of users. 

First, the population estimates of the Statistic Bureau of Ministry of Internal Affairs and Communications for the population nation-wide of persons over 65 years of age and of persons over 75 years of age were used [31,32,33,34,35]. Next, data was collected from the Ministry of Health, Labor and Welfare (MHLW) survey on expenses for long-term care concerning the number of persons needing and certified for elderly support and care; the number of persons receiving preventive care services; and the number of persons receiving nursing care services. This data was collected after the year 2000, when Long Term Care Insurance for the elderly was instituted. It was collected for five years from the year 2002, when the current statistical information format was determined, 2005, 2010, 2013, and 2014 [36,37,38,39,40,41,42,43,44,45]. Furthermore, after 2006, services were divided into preventive care services and nursing care services. Data on the total number of facility users also comes from the same survey. As well, data on the number of users per insurer of elderly care insured facilities came from a survey by the Ministry of Health, Labor and Welfare of each insurer’s elderly care service facilities [46]. However, this Ministry’s survey did not give any statistics regarding the number of users per insurer of community-based services.

Then, in regard to group living facilities for the elderly with dementia (hereafter GHs), and community-based multi-care facilities (hereafter, “CBMCs”), the 5th Term Plans for Elderly Long Term Care Insurance were sampled and extracted from insurers’ web-sites. These plans were taken from insurers, such as municipalities or groups of small municipalities. As of December 2014, there were 1587 insurers nation-wide. The period for this data was integrated with the most recent national census in 2010.

Next, based on this data, the required index values that were needed to calculate the number of facilities and service users were computed. Specifically, the following data were calculated: the ratio of the number of persons certified for care to the number of elderly; the proportion of latter stage elderly (age 75 years or over) among persons certified for care; the proportion of latter stage elderly among persons receiving elderly care services; elderly care welfare facilities (so-called nursing homes for the elderly, hereafter, “nursing homes”); geriatric health services facilities (hereafter ”long-term care health facilities”); GHs; and, CBMCs. The values that were calculated for the above were the user rates of each facility’s services. Section 4 gives a definition of “user rates”.

Furthermore, the 2025 reform model, as shown by the Social Security Council, is pattern 1 of the reform scenario model in the “discussion points related to a review of the nursing care field in Social Security and Tax Integration Reform (2014)” (Table 3) [26]. Reform scenario 1 is formally calculated in accordance with assumptions being based on the current projection scenario [47].

There will be an increase of 140 thousand persons in the number of users of long term care insurance due to a reduction in the number of persons entering hospitals by 2025. 60% of them are expected to use care service facilities. The number of receivers of long-term care insurance will decrease overall by 3% due to the effects of preventive elderly care services and services to prevent a worsening of their condition. Thus, the number of users should decrease from 6.47 million in the current projection to 6.41 million in reform scenario pattern 1.

Facility service: The number of users of nursing homes and long-term-care health facilities is estimated as follows. First, institutional users are estimated for different types of care level and households. The ratio of institutional users to elderly certified as needing support or care is assumed to decrease by 5%. Next, nursing homes and long-term-care health facilities are assumed to be only for severely ill elderly users because nursing homes are facilities for intensive care for the severely ill and long-term care health facilities are for transitional facilities for users going from hospitals to back home. The severely ill elderly users are allocated to nursing homes and long-term-care health facilities in accordance with the current rate of users in each institution for different care levels. Also, 70% of nursing homes and 50% of long-term-care health facilities are expected to introduce care service units to improve the content of care. Thus, nursing homes residents should decrease from 860 thousand in the current projection to 720 thousand in scenario pattern 1. Also, residents of long-term-care health facilities, including medical facilities, should decrease from 750 thousand in the current projection to 590 thousand.

Residential service: GHs for the elderly with dementia are expected to increase as the number of the elderly with dementia increases. Specifically, GHs are expected to continue to be established in accordance with the average growth rate of the past three years. The percentage of GHs residents who have dementia but do not use institutions will increase to 20% (currently 12%), and residents of GHs should increase to 370 thousand in reform scenario pattern 1.

In-home care giving service: Services supporting home living for severely ill elderly persons are expected to be enhanced. To do this, enough CBMCs should be established to handle 4.0 million people per day. An increase of GHs and CBMCs will enhance support for the elderly with dementia.

Based on these, the establishment rate objectives for the facilities were calculated. Regarding the numerical establishment objectives for 2025, the objectives for long-term care health facilities and sanatorium type medical care facilities for the that are elderly requiring care have been totally calculated and analyzed in the following section (as of 2006 there has been a plan to abolish the distinction between these) (Appendix A Note 1). Hereafter, we have abbreviated ‘sanatorium type medical care facilities for the elderly requiring care’ as “medical care facilities”.

Furthermore, in this paper, evaluations have been made by applying the user rate that was calculated from the number of users in 2010, when comparisons with the numerical establishment objectives for 2025 were made. As an alternative, there is a method based on estimation of the number of cases of elderly people needing support or care (Appendix A Note 2). 

### 2.2. Case Research Methods and Analytic Viewpoints

The case research focused on K City in Ishikawa Prefecture where the quantitative situation of an insurer was investigated. Also studied was the possibility of applying to K City the establishment rate objectives for insured elderly care facilities calculated for it. K City was chosen for a case study for two reasons. Firstly, as we see later, because the amount of facilities established is more than sufficient for the number of users and there was accurate data on the number of persons on waiting lists in 2013, K City can be considered as an example from which the number of persons on waiting lists and the number of all the users can be grasped (Appendix A Note 3). Furthermore, in K City in 2013, there were zero beds in long-term-care health facilities, including medical care facilities. Secondly, we could obtain data on different types of households. This is valuable information because there are no nationwide data on them.

In K City, there were 20,714 elderly persons (aged 65 or older) out of a population of 71,611 in April 2013 [48]. The percentage of the total population over the age of 65 years was 28.9%. There are seven care service areas in the city. Trends in the city are the same as those that are nation-wide. That is, that the total population will decrease and the population of persons 65 years or over will increase. 

From K City, data obtained included the sex of persons 65 years or older; their ages; and their type of household. Data was also obtained on the sex of all persons certified as needing elderly care; on their ages; on the extent of care needed; on their type of their household; and, on whether or not they require elderly care services (all data obtained was as of 1 April 2013).

## 3. The Nationwide Proportion of Persons Receiving Elderly Care Services and the Persons Certified as Needing Support or Care

**Figure 2:**
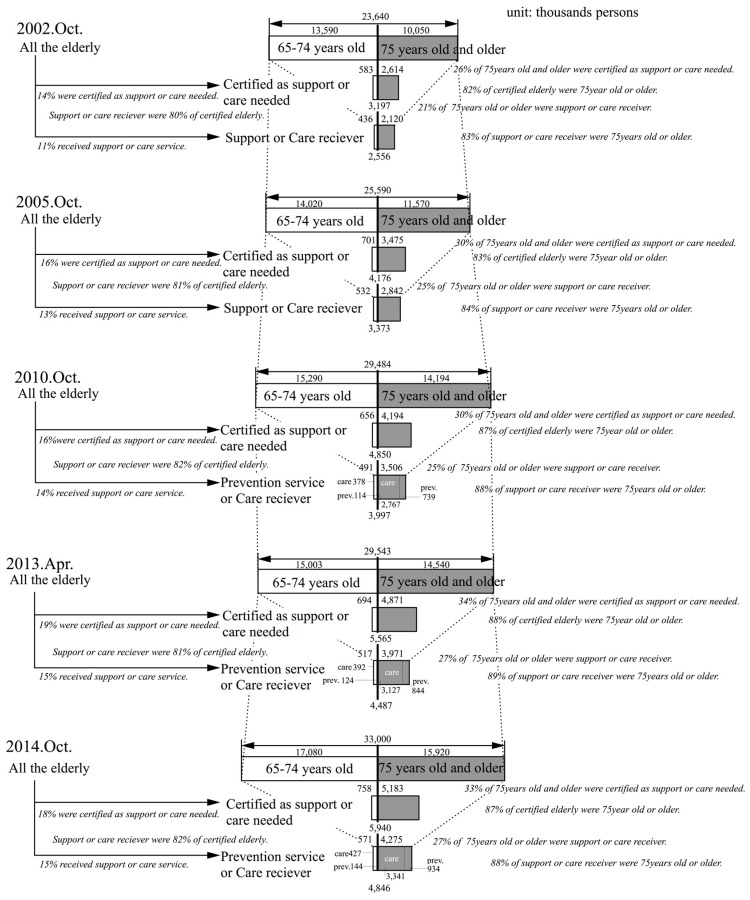
Total number of elderly, certified, and receiver of care services in nationwide Japan (2002, 2005, 2010, 2013, and 2014 in chronological order).

In this section, the proportion nation-wide of persons receiving elderly care services and persons that are certified as needing support or care was determined. Figure 2 shows, in chronological order, the total number of elderly in the years after introduction of this insurance—for the years 2002, 2005, 2010, 2013, and 2014, after long-term care insurance was introduced in 2002. It also shows, in chronological order, the number of persons 65 to 74 years old and 75 years or older who are certified as needing care, and the number of persons receiving preventive care and nursing care services.

First of all, the ratio of the number of persons 75 years or older to the total number of all elderly persons has tended to show a slight increase in the covered period. If the medium-range projections of the National Institute of Population and Social Security Research [25] are looked at, then this number will peak at 62.08% in 2031, then decline and hit bottom in 2042. After that, it will again increase until 2060, the last year for which projections have been made. In other words, the proportion of persons 75 years or older out of the number of persons 65 or older is not a fixed ratio.

When looked at, the proportion of persons 75 years or older who are certified as needing support or care, out of the total number of elderly persons certified as needing support or care, this proportion rose from 82% in 2002 to 88% in 2013, and then it fell slightly to 87% in 2014. Furthermore, the study showed that the proportion of persons 75 years or older who are receiving preventive care services or nursing care services, rose from 83% in 2002 to 89% in 2013, and then it also fell slightly to 88% in 2014. In other words, as of 2014, almost 90% of the elderly persons requiring support or care and 90% of the persons receiving preventive care or nursing care services were 75 years old or older. It is necessary to look closely at these numbers in terms of them rising and coming to a halt in the future.

From the above, the following points can be made. Currently, persons 75 years or older account for about 90% of the persons receiving preventive elderly care and nursing care services. Next, the proportion of persons who are 75 years or older in the elderly population of persons 65 years or older is not necessarily constant. Therefore, when estimating the number of users of care services, it is relatively easy to calculate these numbers by using the population of persons 75 years or older. When looking at the National Institute of Population and Social Security Research’s medium-range projections on population trends for persons 75 years or older [25], it can be seen that in 2030 this number will peak at 22,784,000 persons, the largest number of this population group in Japanese history. Then, after declining slightly to 22,011,000 persons in 2041, it will increase again to a new high of 24,079,000 persons in 2053. Consequently, it is estimated that the periods before and after the years 2030 and 2053, will be peak periods in the demand for elderly care services.

Meanwhile, the ratio of persons that are certified for elderly care among those 75 years or older rose from 26% in 2002 to 34% in 2013, and then it fell slightly to 33% in 2014. Furthermore, the ratio of persons receiving support or care among those 75 years or older rose from 21% in 2002 to 27% in 2013, and then it stayed almost the same at 27% in 2014. The ratio of persons receiving preventive care or nursing care services out of the persons certified as needing support or care was, according to data from 2002 to 2014, almost constant at about 80% (Appendix A Note 4).

The ratio of persons among those age 75 years or over who were receiving care at Level 3 or above was almost constant at 40% (Table 4). The proportion of those 75 years or older receiving care at Level 3 or more increased from 2002 to 2014. 

## 4. User Rate of Nursing Homes, Long-Term Health Care Facilities, GHs, and CBMCs by Nation-Wide Insurer

**Figure 3:**
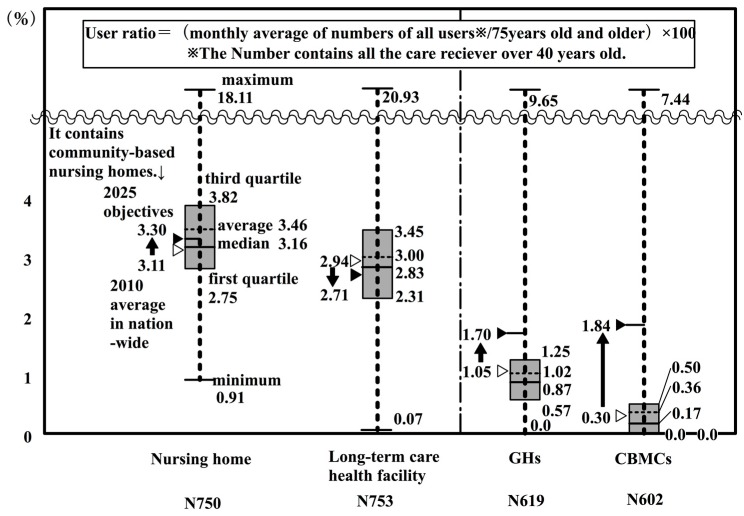
User rate for persons 75 years or older in nationwide insurers’ nursing homes, long-term health facilities, GHs, and community-based multi-care facilities (CBMCs).

In this section, indices are determined for the number of users of long term care insurance related facilities in 2010, by nationwide insurers. Figure 3 expresses in a box plot the user rate for persons 75 years or older in nationwide insurers’ nursing homes, long-term health facilities, GHs, and CBMCs (as of 2010) (Appendix A Note 5). The user rate was calculated as the total number of users of facilities per twelve months divided by the population of persons 75 years or older. Here, the number of users is the total number of primary insured persons (65 years and older) and secondary insured persons (40 to 64 years old). Shown in a box plot on the left of the figure, is the average of user rate according to the type of facility, as calculated from the MHLW’s monthly survey of long-term care expenses [38] and the Ministry of Internal Affairs and Communications’ population estimates [33]. In this way, each insurer can compare their situation with that of other insurers, and with the targeted establishment rate for 2025, which will be discussed later.

There were 420,000 persons on waiting lists for nursing homes in 2009 [49]. Long-term care health facilities and GHs are almost completely full. From this, it can be said that the user rate for these three kinds of facilities can be regarded as almost equal to the establishment rate. However, because there are persons waiting to get into nursing homes, it should be noted that the user rate does not indicate all of the demand for care facilities, such as potential demand.

The national average and the average value of subjected insurers are 2.94 and 3.00 for long-term-care health facilities (including medical care facilities), respectively; 1.05 and 1.02 for GHs, respectively; and, 0.30 and 0.36 for CBMCs, respectively. These values can be regarded as practically within the margin of error. However, in regard to nursing homes, the difference between the national average (3.11) and the average value of insurers (3.46) is large.

This calculation method can produce disparities in the results for the number of users, because the number of users in the numerator is the total annual number of users divided by 12 months.

Next, the facility types were examined. The boxes from the second quartile to the third quartile are, in descending order: nursing homes; long-term health care facilities (including medical care facilities); GHs; and, CBMCs. However, CBMCs are in a lower position because they were introduced in 2006, and as of 2010 they were still in the developing stage. The development of CBMCs should be monitored in the future.

## 5. Comparison of the Establishment Objectives in the 2025 Reform Model with the 2010 National Average

In this section, indices were created for the targeted number of long term care insurance related facilities in the 2025 reform model and compare these with the 2010 national average. To calculate the establishment rate objectives for 2025, the prospective number of users of elderly care facilities was extracted from the article “Issues related to a review of the system of the elderly care in regard to social insurance and tax integration reform” [26] (Table 5).

Using reform scenario pattern 1, the establishment rate objectives were calculated by dividing the number of establishments by the population of 75 years or older in the medium range estimates by the National Institute of Population and Social Security Research [25]. The establishment rate objectives for 2025 were 3.3% for nursing homes; 2.71% for long-term care health facilities (including medical care facilities); 1.7% for GHs; and, 1.84% for CBMCs. If we multiply these indices by the estimates of the population of 75 years or older in 2025 for each insurer, we can get a rough estimate of the establishment objectives.

A comparison can now be made between the nationwide average values in 2010 and the establishment rate objectives for 2025. One point that needs to be given careful consideration is that the denominator in this calculation, that is, the population of persons 75 years or older, will increase by 1.53 times (from 14,194,000 in 2010 to 21,786,000 in 2025 [25]). Even if we suppose that the number of facilities, the numerator in the equation, is maintained as it is, then the establishment rate/population ratio will fall to 0.65 (1/1.53).

First of all, in regard to GHs and CBMCs, the establishment rate objective has been rising from the 2010 nation-wide average. In reform scenario 1, the assumption is that home services and residential services will expand. This assumption is backed up by the setting of objectives of very large increases in these services. Specifically, in Table 5, the amount for GH establishment objectives will increase by 2.3 times from 2010 to 2025 in reform scenario 1. For CBMCs, the objective for these facilities is an increase by 8.1 times in the quantity of these services that are currently provided. In other words, we can see that along with the prospective absolute increase in the number of users of GHs and CBMCs, the establishment rate objectives for these facilities will increase as well.

Next, for long-term-care health facilities (including medical care facilities), the national average of 2.94% in 2010 will fall to an establishment rate objective of 2.71% for 2025, a relative decrease of 0.92%. If we also look at the quantitative establishment objectives in Table 5, it will be held to 1.3 times, although user rate will be 1.5 times.

Finally, in regard to nursing homes, data generated by insurers for community-based nursing homes has not been made public. So, the nursing home user rate does not include the users who are in community-based nursing homes. On a nation-wide level, the average for 2010 including community-based nursing homes is 3.17% (Appendix A Note 6). Furthermore, it became 3.47% in 2013, and the establishment rate objective for 2025 will slightly increase to 3.3%. If we look at the current projections in Table 5, the amount for targeted establishment has become 1.8 times greater, when compared to a 1.5 times increase in the user rate. However, when the same numerical values are converted into an establishment rate objective, the establishment rate objective becomes 3.95%. When compared to the national average of 3.17%, it stays at 1.2 times. Next, the number of users in the nursing home establishment objective in the reform scenario pattern 1 is almost 1.5 times the current number. When the same value is converted into an establishment rate objective, it becomes 3.30%. In regard to the 2010 nation-wide average of 3.17%, it is 1.04 times, which means that the current situation is going to be kept.

From the above, when the 2010 nationwide average values are compared with the 2025 reform model establishment rate objectives, it can be seen that GHs and CBMCs will raise their establishment rate objectives, along with an increasing quantity in regard to the prospects of an increase in the absolute number of users of GHs and CBMCs. On the other hand, the establishment rate objective for nursing homes will be almost the same as it is currently. However, long-term health care facilities will instead be suppressed.

## 6. Grasping the Quantitative Situation for Support or Care-Requiring Elderly for an Insurer, K City, and Studying the Possibility of Applying the 2025 Establishment Rate Objectives

In this section, the study looks at an insurer, K City, in the Ishikawa Prefecture, specifically evaluating the quantitative situation for the support or care-requiring elderly, and studying the possibility of applying the 2025 establishment rate objectives. According to the medium-range estimates by National Institute of Population and Social Security Research [25], the population of persons 75 years or older will be at a maximum of 14,109 in K city in 2025.

### 6.1. Proportion of Persons who are Certified as Needing Support or Care, and of Persons Receiving Elderly Care Services

(1) Proportion of persons who are certified as needing support or care, and of persons receiving elderly care services

**Figure 4:**
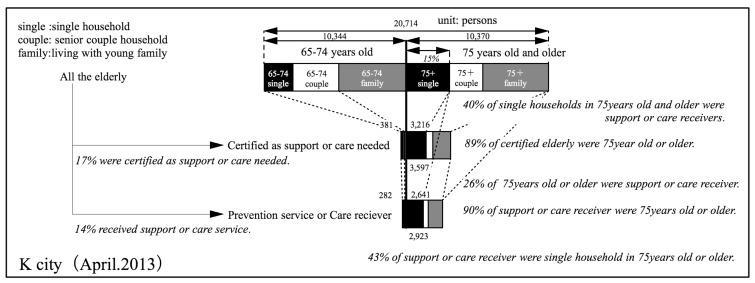
Total number of elderly, certified, and receivers of care services in K city.

First, we created a figure (Figure 4) that was the same as Figure 2 for the current situation in K City as of April 2013. In Figure 4, the types of households are also given. In K City, among all of the elderly, 14% are persons who receive some type of care service. Among the support or care-requiring elderly and persons receiving preventive services or care services, 90% are aged 75 or older. This number is almost the same as that of elderly care services nation-wide.

(2) Relation of type of household and receipt of elderly care services

The types of households can be seen in Figure 4. Single householders 75 years or older comprise about 15% of the elderly population. Among these, about 40% are persons that are receiving preventive care or care services. On the other hand, among households of couples who are 75 years or older, about 15% are persons receiving such services. Among persons living together with family members, 22% were persons that are receiving such services. In other words, when the types of households for persons aged 75 or older were looked at, the elderly care services rate for persons from single-person households was higher than that of persons from elderly-couple households or households including other family members. This suggests that the composition of households will affect the demand for services.

Of the total number of persons 75 years or older receiving preventive care and elderly care services, 43% are living alone; 12% are couples; and, 36% are living with other family members. In other words, about half the number of people 75 years or older are living with family members, and they account for about 40% of care recipients.

Here the reasons why the rate for receiving senior care services is so high for single person households are considered. First, a reason posited is the fact that in a single elderly person’s household there is no one in the person’s family who is able to act as a caregiver.

**Figure 5:**
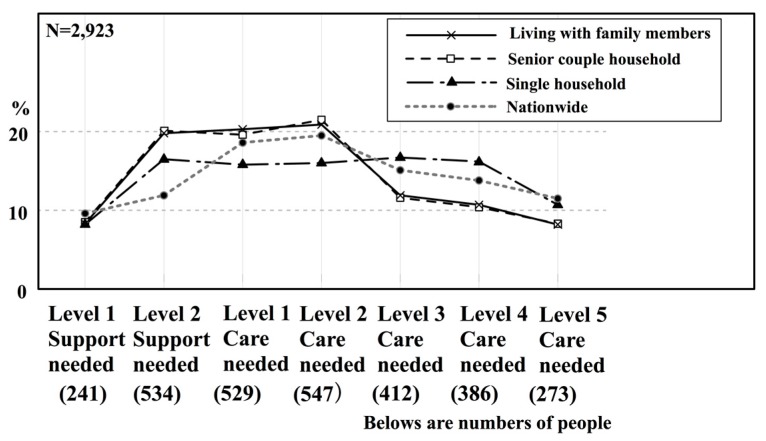
Composition of the need for elderly care according to household type in K City who are 65 years or older and are receiving care (Nationwide data is from [50]).

The second reason offered is the fact that in K City the ratio of persons in a severe condition who need elderly care is somewhat high among single person households. Figure 5 shows the composition of the need for elderly care, according to household type for persons in K City who are 65 years or older and are receiving elderly care. In K City, single-person households needed about the same proportion of Level 2 support as Level 4 care.

When compared with nation-wide values for the same period, Level 2 support needed is higher, Levels 1–2 care needed is slightly lower, and Levels 3–4 care needed is slightly higher.

In other words, in K City, the proportion of persons in a single person household who are in a severe condition is slightly higher than for the proportion of these persons nation-wide. The lines for senior couple households and for households living with family members slightly overlap. They are almost the same as the lines for nation-wide values. As compared to nation-wide values, the proportion of persons that needed Level 2 support is quite high, and the proportion of persons that needed Levels 3–5 care is slightly lower. In other words, in K City, the proportion of persons from senior couple households or households living with family members who require a slight degree of elderly care is higher than the proportion nation-wide.

From the above, two reasons can be expanded as to why the rate of receiving elderly care services is high for single person households. The first is that, in single person households, there is no family member who can provide caregiving. The second is that, in K City, among elderly single person households, the proportion of persons who are in a severe condition and need care is high.

**Figure 6:**
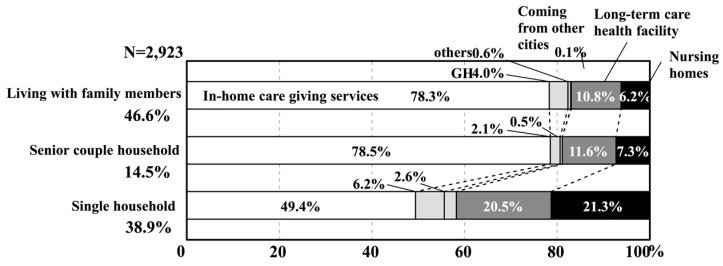
Proportion of services used according to type of household where the person is aged 65 years or older in K City.

**Figure 7:**
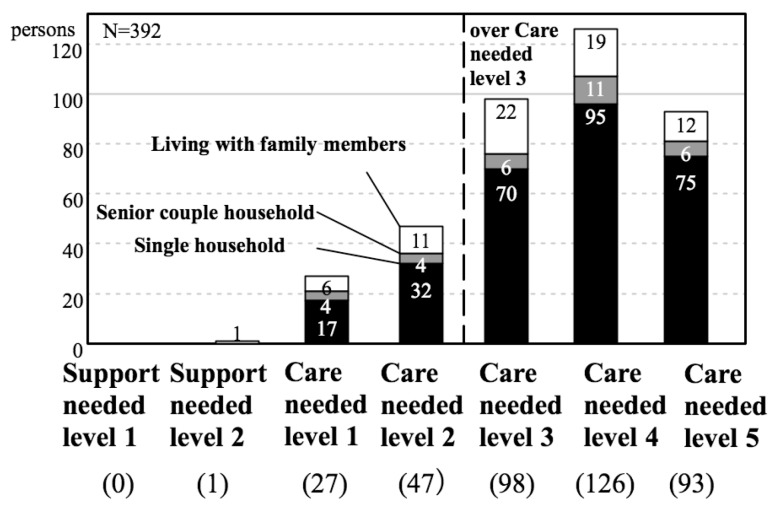
Number of persons aged 65 years or older in K City in nursing homes according to the severity of condition and household type.

Then, what kind of elderly care services have single person households used? Figure 6 shows the proportion of services (in-home care giving services or various facilities services) that are used according to the type of household for persons aged 65 years or older in K City. For households living with family members and for senior couple households, home services account for approximately 80% of the services used. On the other hand, for single person households, home services account for about 50% of the services used, and the proportion of persons in nursing homes and long-term care health facilities is clearly high. Furthermore, Figure 7 shows the number of persons aged 65 or older in K City in nursing homes according to the severity of the condition and according to household type. No matter what level of severity of the condition, single person households account for more than half of the rate. Reiterating the reasons for these situations that are given above, it can be extrapolated that in single person households, there is no family member available to provide caregiving services. This suggests the possibility that the type of household will also affect the demand for institutional services.

### 6.2. The Establishment Rate and User Rate for Nursing Homes, Long-Term Care Health Facilities, GHs, and CBMCs

Next, the study looked at the user rate in regard to the population of persons 75 years or older in nursing homes, long-term care health facilities (including medical care facilities), GHs, and CBMCs.

(1) Capacity for each facility and the actual number of users

**Figure 8:**
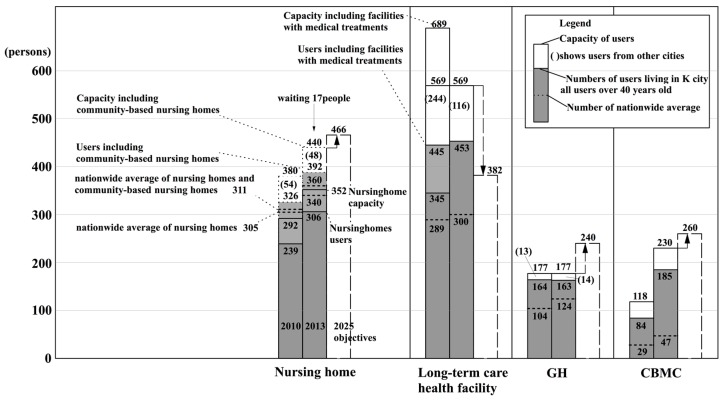
Actual situation regarding the capacity and the number of users for each type of facility in K city.

Firstly, Figure 8 shows the actual situation regarding the capacity for each type of facility and the number of users (for all of the recipients of primary and secondary insured persons). At a glance, it can be seen that the number of users of long-term care health facilities, GHs, and CBMCs, is greater than the nation-wide mean reduced value in 2010. From this, it is known that there are sufficient facilities being provided. On the other hand, for nursing homes, the national mean converted value includes users of nursing homes and community-based nursing homes. In 2010, when compared to the national mean converted value of 311 users, K City had 326 users. In 2013, as compared to the national mean converted value of 360 users, the number for K City increased to 392 users. Furthermore, as of 2013, there were 17 persons who met the criteria for entering a nursing home and were on a waiting list for entering a nursing home (Appendix A Note 7). From this, it can be said that K City is an area where there is a large number of users of nursing homes.

(2) Establishment rate and user rate according to type of facility

**Figure 9:**
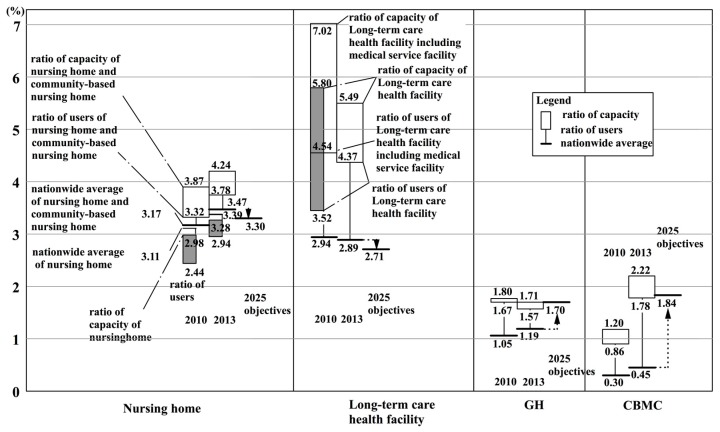
User ratio and establishment objectives on nursing home, long-term care health facility, group living, and community based multi-care facility in 2010, 2013 and 2025.

Next, the study looked at the establishment rate according to type of facility and user rate (Figure 9).

Firstly, the user rates of GHs are 1.67% (2010) and 1.57% (2013), and these rates are higher than those for the national averages of 1.05% (2010) and 1.19% (2013). When compared with Figure 3, the 2010 values surpass the third quartile of nation-wide insurers. The rate of 1.71% for establishment has almost achieved the nation-wide establishment objective of 1.7% for the year 2025. However, that does not mean that future facilities will not need to be established, because the population of persons aged 75 years or older, the numerator of the equation, is expected to increase by 2025.

Next, the user rates for CBMCs were 0.86% (2010) and 1.78% (2013). These rates were both above the national average of 0.30% (2010) and 0.45% (2013). The value for 2010 surpasses the third quartile for nation-wide insurers in Figure 3. The 2013 establishment rate of 2.22% surpasses the nation-wide establishment objective of 1.84% for the year 2025.

**Figure 10:**
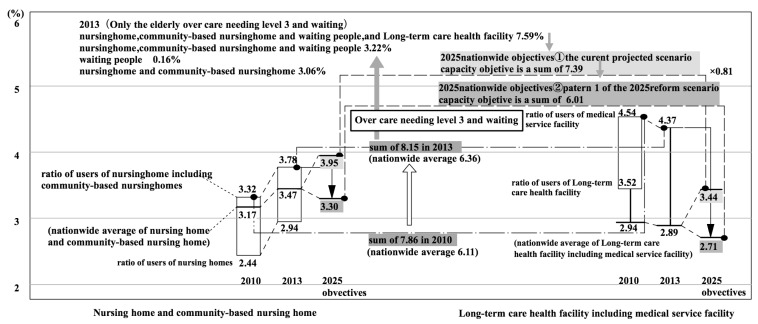
User ratio and establishment objectives on nursing home and long term care health facility (extracted from Figure 9 and expanded).

Furthermore, Figure 10 expands on parts of the nursing homes and long-term care health facilities shown in Figure 9. In regard to the population of persons aged 75 or over, the total rates of users of nursing homes and community-based nursing homes were 3.32% (2010) and 3.78% (2013). Both of these rates were higher than the national averages of 3.17% (2010) and 3.47% (2013). In Figure 3, the values for 2010 are between the median and the third quartile for nation-wide insurers.

According to K City, there have been cases in which persons who were on a waiting list for a nursing home entered a long-term care health facility. However, when their turn came up to enter the nursing home, they continued to stay in the long-term care health facility. In other words, the actual situation is that long-term health care facilities can be seen as providing substitutes for nursing home, although they are supposed to function as intermediate facilities. Among persons in long-term care health facilities, the proportion of persons who would be better suited to nursing homes is unclear. So, the user rate for nursing homes cannot be calculated in a way that would include persons that are better suited to nursing homes than to long-term care health facilities (Appendix A Note 8).

For the sake of convenience, if we total the user rate for nursing homes and long-term care health facilities (including medical care facilities), then the user rates are 7.86% (2010) and 8.15% (2013). The total values nation-wide were 6.11% (2010) and 6.36% (2013). In other words, the total user rates in K City for nursing homes and long-term care health facilities were considerably larger than the national averages. One factor that can be pointed out in this difference is that the user rates for long-term care health facilities (with medical care facilities) were 4.52% (2010) and 4.37% (2013), which were considerably above the national average of 2.94% (2010) and 2.89% (2013). When considering the value for 2010 it could be located above the third quartile in Figure 3.

### 6.3. The Relationship Between the User Rate for Nursing Homes and Long-Term Care Health Facilities and the Establishment Rate Objectives for 2025

In this study, the 2025 establishment rate objectives are compared with the user rates in 2010 and 2013 for nursing homes and long-term care health facilities (Figure 9 and Figure 10). Firstly, the rates for long-term care health facilities are compared showing that in regard to the 2013 establishment rate of 5.49% and the user rate of 4.37%, the targeted establishment rate under the 2025 reform scenario 1 is 2.71%. In this scenario, the establishment level is lowered considerably.

Next, comparisons for nursing homes are made. The following three points must be considered. The first is that the system was changed in April 2015 so that an elderly person must now require Level 3 or above care to be accepted into a nursing home (Appendix A Note 9). When this change is reflected in the user rate, the user rate becomes 3.06% among users of nursing homes or community-based nursing homes with respect to the population of persons aged 75 years or more. On the other hand, among the users of nursing homes (including community-based nursing homes) in K City, users requiring Level 3 care or more account for 80.8% of the total (Table 6). This is slightly below the value for Japan as a whole. In other words, the proportion of persons in nursing homes who require less than Level 3 care in K City is slightly greater than the national average.

The second point concerns persons on waiting lists for nursing homes. There were persons on waiting lists for nursing homes in K City. When the portion of 17 persons (0.16%) on waiting lists are included with the user rate given above, 3.06%, the total becomes 3.22%. This rate is almost the same as that of the 3.3% nursing home establishment objective in pattern 1 of the 2025 reform scenario.

The third point is the use of alternatives to nursing homes. As discussed above, as a matter of convenience, the user rates for nursing homes and long-term health care facilities were combined. The reason for doing this was that accurate data was lacking on the number of persons who were in long-term health care facilities because they could not get into nursing homes. When the user rate for long-term healthcare facilities was added to the 3.22% above for nursing homes, the result was 7.59% (Appendix A Note 10). This value is almost the same as that of the rate conversion total of 7.39% for the targeted establishment for nursing homes and long-term health care facilities in the current projected scenario for 2025.

In other words, the total user rate for nursing homes and long-term health care facilities in K City in 2013 was almost the same as the establishment rate objective total for nursing homes and long-term health care facilities in the current projected scenario for 2025. (The total user rate for K City in 2013 only included the elderly in care requiring Level 3 or greater and persons on waiting lists). However, to accord with pattern 1 of the 2025 reform scenario, it needs to be reduced to 80%.

### 6.4. A Study of the Possibility of Applying the 2025 Nation-Wide Establishment Rate Objectives

Finally, the establishment rate objective of pattern 1 of the reform scenario for 2025 was applied to K City, and a study was made of its possible application (Figure 8, Figure 9 and Figure 10).

The quantified establishment objectives for 2025 were easily calculated by multiplying the establishment rate objectives by the estimated population of persons 75 years or older in K City in 2025 (14,109 according to medium range estimates of the National Institute of Population and Social Security Research [25]). The results were as follows: 26 more beds were needed for nursing homes; 187 fewer beds were needed for long-term-care health facilities; 63 more beds were needed for GHs; and, 30 more members were needed for CBMCs. Like above, by using the establishment rate objectives, the establishment target in quantitative terms for scenario 1 of the 2025 reform model could easily be calculated.

Some problems that were related to application of the targeted establishment rate need to be addressed. The first problem concerns the existence of persons whose former address is outside of K city. In other words, this problem is related to the adjustment of supply and demand between insurers. In Figure 8, it can be seen that the quantities for establishment according to the type of facility are necessarily greater than the number of users, but there were no vacant beds in the nursing homes, long-term care health facilities, and GHs. The disparity between these capacities and the number of users in K City is due to the fact that residents of other cities have entered into these facilities. Therefore, for the application of the establishment rate objectives in the case of nursing homes, there is a limitation to calculation using only a single city or insurer (Appendix A Note 11). A broader framework, such as prefectures and regions, needs to be looked at.

The second problem concerns the entrance into long-term care health facilities as an alternative by persons on a waiting list for entrance into a nursing home. In other words, it concerns the adjustment of supply and demand between different types of facilities. There is no information available on the number of persons in long-term care health facilities who are waiting to enter nursing homes, nor is it known whether it is appropriate for them to be in these long-term care health facilities. In Section 6.3, as a matter of convenience, a method of calculating for the user rate, the total number of people in nursing homes, in long-term care health facilities, and on waiting lists was adopted.

Changes occurring from now on should be considered for further study. These would include changes in the number of persons certified as needing support or care services and changes in the number of persons that are receiving preventive or elderly care services. Looking at these changes, it would be considered appropriate to study whether each index can be stabilized. It is also predicted that there will be an improvement in the user rate of CBMCs, which were not supplied very much in 2010.

Furthermore, this study attracted suggestions that the type of composition of a household influenced estimates of demand for services, but the connection between these suggestions and the calculation of any specific user rate was not made. The author also would like to set up as an issue for future study an investigation into the adjustment of demand among different insurers and among different types of facilities.

## 7. Conclusions

In this research, the quantitative situation of care-requiring elderly people in Japan was investigated. The study also determined and indexed the number of users of elderly care according to the type of care insurance facility and according to nation-wide insurers in 2010. In addition, the quantities for establishment objectives for the elderly facilities shown in the government’s reform model for 2025 were indexed, and the user rates were compared. In addition, K City was studied and the possibility of applying the targeted establishment rates for 2025 was investigated.

As a result, the establishment rate objectives (with respect to persons aged 75 years or older) in the 2025 reform model were calculated, the results were 3.3% for nursing homes; 2.71% for long-term care health facilities; 1.7% for GHs; and, 1.84% for CBMCs. When comparing the rates of the users in the 2010 national average with those of the establishment objectives in the 2025 reform model, it could be seen that the establishment rate objectives for GHs and CBMCs will increase in conjunction with a prospective increase in the absolute number of users. Establishment rate objectives for nursing homes will stay the same, and the establishment rate objectives for long-term care health facilities will be suppressed. They reveal that the Japanese social care system is shifting to realize ‘Ageing in Place’. Japan is now in phase 3 of Hiroi’s model, and it is facing the difficulty of changing from an institution centered system to an in-home care-giving service model, which realizes the concept of ‘Ageing in Place’.

Based on a case study in K City, the rate of receiving services is higher for single person households than for senior couple households or households living with family members. As an implication for long-term care policy and practice, household types need to be considered. Institutionalizing services are popular for single-person households, whereas in-house care-giving services are popular for elderly-couple households and households containing other family members.

There are also problems related to persons who are on a waiting list for nursing homes and enter long-term care health facilities as an alternative. These problems stem from insufficient adjustment of demand between types of facilities. To better balance demand and supply, it is worth thinking about converting long-term-care health facilities into nursing homes, because the former substantially play the same role as the latter.

Also, when considering households tendencies, these target ratios for the establishment of facilities are supposed to be applicable to other countries in Asia. Especially, early establishment of GHs is expected to be efficient both to properly care for the elderly with dementia and to smoothly shift to the in-home caregiving service model in the future.

## Figures and Tables

**Table 1:** Community-based Integrated Care System (information from Ministry of Health, Labor and Welfare (MHLW) [23]).

	Key Provisions	Provider	Finance
Housing	One’s own residence, elderly residences	Private sector, authorized social welfare organization	-
Medical service	Regular healthcare at hospitals and clinics	Authorized medical organization	Medical insurance
Long-term care	In-home service, long-term care service, facility residence service (details in Table 2)	Authorized social welfare organization.	Long-term care insurance
Preventative long-term care	In-home service, preventive long-term care service (details in Table 2)	Authorized social welfare organization	Long-term care insurance
Livelihood support	Exercise, watching over	Seniors’ club, residents’ association, volunteer group, NPOs, etc.	-

**Table 2:** Detailed menu of long-term care and preventive long-term care services [24].

Services Covered by Long-Term Care (LTC) Insurance	Percentage of Expense
Home-based services (including nursing care preventive services)	49.3
Home-visit/outpatient	38.6
Home-visit long-term care	9.8
Home-visit bathing service	0.6
Home-visit nursing care	2.1
Home-visit rehabilitation	0.4
Daycare for long-term care	17.6
Daycare rehabilitation	5.1
Rental of assistive equipment	3
Short-term institutionalization	5
Short-term stay for LTC	4.4
Short-term stay for recuperative care (in LTC health facilities)	0.6
Short-term stay for recuperative care (in hospitals, etc.)	0
Home-based recuperative care control and instruction	0.8
LTC for residents of specified institutions (excluding short-term use)	4.9
LTC for residents of specified institutions (short-term use)	0
In-home care support services	5
Community-based services	11.6
Periodic or on-call home-visit LTC and nursing care	0.2
Nighttime home-visit care	0
Daycare for LTC of elderly with dementia	0.9
Small-scale multifunctional home-based LTC	2.1
LTC for the elderly with dementia in residential care settings (excluding short-term use)	6.5
LTC for the elderly with dementia in residential care settings (for short-term use)	0
Community-based LTC for residents of specified facilities (excluding short-term use)	0.2
Community-based LTC for residents of specified facilities (short-term use)	0
Community-based LTC in welfare facilities for elderly requiring LTC	1.5
Combined services	0.1
Service in institutions	34.1
Facility covered by public aid providing LTC to elderly (nursing home)	17.6
LTC health facility	13.2
Sanatorium-type medical care facilities for elderly requiring care	3.2

**Table 3:** Prospective number of users of elderly care facilities from “Issues related to a review of the system of the elderly care in regard to social insurance and tax integration reform” [26].

	2011	2025 Current Projection Scenario (Magnification)	2025 Reform Scenario Pattern 1 (Magnification)
Number of users	4.26 million	6.47 million (1.5 times)	6.41 million (1.5 times)
Facility service; Nursing homes including community-based nursing homes	480 thousand	860 thousand (1.8 times)	720 thousand (1.5 times)
Facility service; Long-term-care health facilities including medical facilities	440 thousand	750 thousand (1.7 times)	590 thousand (1.3 times)
Residential services; Group living	160 thousand	270 thousand (1.7 times)	370 thousand (2.3 times)
In-home care-giving services; Community-based multi-care facilities	50 thousand	80 thousand (1.6 times)	400 thousand (8.1 times)

**Table 4:** Ratio of persons among those age 75 years or over who were receiving care at Level 3 or above.

Year	2002	2005	2010	2013	2014
Ratio of elderly over care needing level 3 among receiver of care services over 75 years old	40.80%	39.20%	42.10%	40.90%	39.60%
Ratio of elderly over 75 years old among receiver of care services over care needing level 3	80.80%	82.40%	85.60%	86.70%	86.80%

**Table 5:** Prospective number of users of elderly care facilities from the article “Issues related to a review of the system of the elderly care in regard to social insurance and tax integration reform” [26].

	2011	2025 Current Projection Scenario (Magnification)	Establishment Rate Objectives	2025 Reform Scenario Pattern 1 (Magnification)	Establishment Rate Objectives
Number of users	4.26 million	6.47 million (1.5 times)	-	6.41 million (1.5 times)	-
Nursing homes including community-based nursing homes	480 thousand	860 thousand (1.8 times)	3.95%	720 thousand (1.5 times)	3.30%
Long-term-care health facility including medical facility	440 thousand	750 thousand (1.7 times)	3.44%	590 thousand (1.3 times)	2.71%
GHs	160 thousand	270 thousand (1.7 times)	1.24%	370 thousand (2.3 times)	1.70%
CBMCs	50 thousand	80 thousand (1.6 times)	0.37%	400 thousand (8.1 times)	1.84%

**Table 6:** Ratio of users of nursing homes over care needing Level 3 (resource of nationwide data is [51]).

	(A) Number of Nursing Home Users Over Care Needing Level 3	(B) Number of Nursing Home Users	A/B (%)	A/Population over 75 Years Old (%)
Nationwide in 2010	349,114	396,356	88.10%	2.45%
K city in 2013	317	392	80.80%	3.06%

## Appendix A Notes

Note 1: The MHWL announced in 2006 a plan encouraging the abolition of all 130,000 beds in sanatorium-type mal care facilities for the elderly requiring care, and a reduction from 250,000 to 150,000 beds in medical care facilities for the elderly requiring care, and the conversion of the sanatorium-type facilities into long-term-care health facilities. However, as of 2011, they changed the plan again: sanatorium-type medical care facilities can continue under certain conditions until 2017 [52].

Note 2: The amount of demand for elderly care services can be calculated by multiplying the incident rate for certifications of needing support or care services by the elderly population. This amount of demand can be divided with almost no overlapping into demand for the following: in-home caregiving services; CBMCs; GHs; elderly residences; nursing homes; long-term-care health facilities; and medical care facilities. These numbers are all the certified people including those not currently using services. Meanwhile, this research uses the numbers of persons receiving services not including persons on waiting lists or not using services.

Note 3: With the amount of facility capacity more than enough for the number of users, there is sufficient supply with respect to demand. Therefore, it is possible for all persons who wish to use facilities to do so. On the other hand, there is the possibility that there will be an excessive number of users. In such a case, ‘number of users = amount of total demand’ will not be true.

Note 4: According to K City, among persons requiring a small degree of care, there are ‘advanced certified persons’ (persons who are certified as needing services because obtaining approval and certification takes time). On the other hand, in the case of persons requiring a large degree of care, it was thought that a reason for their not using long-term care insurance is that by entering a hospital they switch over to coverage by medical insurance.

Note 5: For the box plot, the value corresponding to 1/4 of the total number indicates the first quartile; the value exactly in the middle indicates the second quartile (the median); and the value corresponding to 3/4 of the total number indicates the third quartile.

Note 6: The number of users of nursing homes and community-based nursing homes was calculated based on data from [38,53] for the years 2010 and 2013. These were calculated by dividing the number of users by the population of persons aged 75 years or more.

Note 7: As of 2013, the number of persons waiting for entering nursing homes was 51. Among these, there were 17 persons who were judged as being in a condition suitable for admission to a nursing home (They had a score of 80 or more on the criteria for entrance into nursing homes designated by Ishikawa Prefecture). In other words, only 1/3 of the persons waiting for nursing homes met the appropriate criteria for admission.

Note 8: According to documents from the MHLW in September 2010, 9.3% of the persons who left a long-term care health facility went into a nursing home [54]. However, this figure does not show the proportion of persons who were on waiting lists for a nursing home or who were supposed to transfer to a nursing home while staying in long-term-care health facilities.

Note 9: According to these regulation revisions, persons who currently require less than Level 3 care and are in a nursing home can live in a nursing home as a transitional measure but cannot enter a new nursing home. For this reason, there is a risk that there will be no place to go for elderly persons who want to enter a nursing home or who require elderly care, live alone in a single-person household, and require less than Level 3 care. (An example: a person who lives alone in a house and rents it).

Note 10: Because the number of persons on waiting lists for nursing homes who were staying at long-term-care health facilities is not clear, this number is considered to contain some duplications.

Note 11: From the point of view of establishing ‘Community-based Integrated Care Systems’, the service area of community-based facilities such as GHs and CBMCs being only inside of a city—junior high school district is an example of this. There should not be cases in which persons living outside the city enter its elderly care facilities. However, nursing homes and long-term-care health facilities cover an ‘elderly health and welfare planning area,’ which is a wider area than a city (it is almost the same as the secondary medical service planning area; for example, there are 4 secondary medical service planning areas in Ishikawa Prefecture). Therefore, a person enters a facility that is not in the area he/she lives.
